# Supplementary material for: Gender disparity in the individual attitude toward longevity among Japanese population: Findings from a national survey
Source: PLoS One. 2021 Aug 18;16(8):e0254779. doi: 10.1371/journal.pone.0254779 (PMC8372936; doi:10.1371/journal.pone.0254779)
Supplement: Attachment — Submitted filename: Response to reviewers.docx [file pone.0254779.s002.docx]

フォームの始まり

PONE-D-20-25313
Gender disparity in the individual attitude toward longevity among Japanese population: Findings from a national survey

PLOS ONE

Dear Dr. Gai,

Thank you for submitting your manuscript to PLOS ONE. After careful consideration, we feel that it has merit but does not fully meet PLOS ONE’s publication criteria as it currently stands. Therefore, we invite you to submit a revised version of the manuscript that addresses the points raised during the review process.

Please submit your revised manuscript by Jan 23 2021 11:59PM. If you will need more time than this to complete your revisions, please reply to this message or contact the journal office at plosone@plos.org. When you're ready to submit your revision, log on to https://www.editorialmanager.com/pone/ and select the 'Submissions Needing Revision' folder to locate your manuscript file.

Please include the following items when submitting your revised manuscript:

- A rebuttal letter that responds to each point raised by the academic editor and reviewer(s). You should upload this letter as a separate file labeled 'Response to Reviewers'.
- A marked-up copy of your manuscript that highlights changes made to the original version. You should upload this as a separate file labeled 'Revised Manuscript with Track Changes'.
- An unmarked version of your revised paper without tracked changes. You should upload this as a separate file labeled 'Manuscript'.

If you would like to make changes to your financial disclosure, please include your updated statement in your cover letter. Guidelines for resubmitting your figure files are available below the reviewer comments at the end of this letter.

If applicable, we recommend that you deposit your laboratory protocols in protocols.io to enhance the reproducibility of your results. Protocols.io assigns your protocol its own identifier (DOI) so that it can be cited independently in the future. For instructions see: <http://journals.plos.org/plosone/s/submission-guidelines#loc-laboratory-protocols>

We look forward to receiving your revised manuscript.

Kind regards,

Thach Duc Tran, M.Sc., Ph.D.

Academic Editor

PLOS ONE

Response to Journal Requirements

When submitting your revision, we need you to address these additional requirements.

1. Please ensure that your manuscript meets PLOS ONE's style requirements, including those for file naming. The PLOS ONE style templates can be found at

https://journals.plos.org/plosone/s/file?id=wjVg/PLOSOne_formatting_sample_main_body.pdf and

https://journals.plos.org/plosone/s/file?id=ba62/PLOSOne_formatting_sample_title_authors_affiliations.pdf

→ The manuscript has been formatted following the suggested templates.

2. As part of your revision, please complete and submit a copy of the RECORD checklist, a document that aims to improve reporting and reproducibility of observational studies that use routinely-collected data for purposes of post-publication data analysis and reproducibility: (http://record-statement.org). Please include your completed checklist as a Supporting Information file. Note that if your paper is accepted for publication, this checklist will be published as part of your article.

→A RECORD checklist has been created as required.

3. Please clarify whether the data utilized in this study were de-identified/anonymised before access?

 →Yes the data utilized in this study were anonymized in the survey.

4. Please revise your discussion and conclusions to emphasize on associations - and not causation/ determinant - between exposure variables and longevity desire. Due to susceptibility to bias and confounding and inability to determine temporal precedence, a cross-sectional study is not the appropriate study design for generating evidence for causation or determinants of outcomes.

 →Related parts have been revised as suggested.

5. Please include your tables as part of your main manuscript and remove the individual files. Please note that supplementary tables (should remain/ be uploaded) as separate "supporting information" files

 →Tables were included in the main manuscript.

6. Thank you for stating the following in the Acknowledgments Section of your manuscript:

[This study is granted by the Project of “The National Survey on Social Security and
People’s Life”.]

We note that you have provided funding information that is not currently declared in your Funding Statement. However, funding information should not appear in the Acknowledgments section or other areas of your manuscript. We will only publish funding information present in the Funding Statement section of the online submission form.

Please remove any funding-related text from the manuscript and let us know how you would like to update your Funding Statement. Currently, your Funding Statement reads as follows:

 [The author(s) received no specific funding for this work.]

Please include your amended statements within your cover letter; we will change the online submission form on your behalf.

→ The statement on funding has been removed.

7. We note that you have indicated that data from this study are available upon request. PLOS only allows data to be available upon request if there are legal or ethical restrictions on sharing data publicly. For information on unacceptable data access restrictions, please see http://journals.plos.org/plosone/s/data-availability#loc-unacceptable-data-access-restrictions.

In your revised cover letter, please address the following prompts:

a) If there are ethical or legal restrictions on sharing a de-identified data set, please explain them in detail (e.g., data contain potentially identifying or sensitive patient information) and who has imposed them (e.g., an ethics committee). Please also provide contact information for a data access committee, ethics committee, or other institutional body to which data requests may be sent.

b) If there are no restrictions, please upload the minimal anonymized data set necessary to replicate your study findings as either Supporting Information files or to a stable, public repository and provide us with the relevant URLs, DOIs, or accession numbers. Please see http://www.bmj.com/content/340/bmj.c181.long for guidelines on how to de-identify and prepare clinical data for publication. For a list of acceptable repositories, please see http://journals.plos.org/plosone/s/data-availability#loc-recommended-repositories.

We will update your Data Availability statement on your behalf to reflect the information you provide.

→ The data used in the study are available upon application to National Institute of Population and Social Security (IPSS), based on relevant regulations of Ministry of Health, Labor and Welfare of Japan on national statistics. Although the authors do not have the authorization on sharing data publicly, the data are available upon application for research purposes. For inquiry or request of the survey data, please contact the link below and mention “The National Survey on Social Security and People's Life 2017”.

http://www.ipss.go.jp/mail/e_sendmail/mail.html

This issue has been added in the cover letter as well.

Response to Reviewers' comments:

Reviewer's Responses to Questions

**Comments to the Author**

1. Is the manuscript technically sound, and do the data support the conclusions?

The manuscript must describe a technically sound piece of scientific research with data that supports the conclusions. Experiments must have been conducted rigorously, with appropriate controls, replication, and sample sizes. The conclusions must be drawn appropriately based on the data presented.

Reviewer #1: Yes

2. Has the statistical analysis been performed appropriately and rigorously?

Reviewer #1: Yes

3. Have the authors made all data underlying the findings in their manuscript fully available?

The [PLOS Data policy](http://www.plosone.org/static/policies.action#sharing) requires authors to make all data underlying the findings described in their manuscript fully available without restriction, with rare exception (please refer to the Data Availability Statement in the manuscript PDF file). The data should be provided as part of the manuscript or its supporting information, or deposited to a public repository. For example, in addition to summary statistics, the data points behind means, medians and variance measures should be available. If there are restrictions on publicly sharing data—e.g. participant privacy or use of data from a third party—those must be specified.

Reviewer #1: No

4. Is the manuscript presented in an intelligible fashion and written in standard English?

PLOS ONE does not copyedit accepted manuscripts, so the language in submitted articles must be clear, correct, and unambiguous. Any typographical or grammatical errors should be corrected at revision, so please note any specific errors here.

Reviewer #1: Yes

5. Review Comments to the Author

Please use the space provided to explain your answers to the questions above. You may also include additional comments for the author, including concerns about dual publication, research ethics, or publication ethics. (Please upload your review as an attachment if it exceeds 20,000 characters)

Reviewer #1: Societies are getting older around the world. Considering this fact, this study examines the anticipation to longevity from a gender perspective using data from Japan. This paper study a very interesting topic, nevertheless some changes may be made to improve it.

GENERAL

1. Including the number of pages and lines would help the revision process.

→ The number of pages and lines have been added.

2. Please, check line by line. Some typos or problems in the word order can be found.

→ Spelling problems have been corrected.

INTRODUCTION

3. I would like to read something referring the characteristics of the Japanese society: the role of women, the role of families, the role older people play in society, the quality of support networks, the general feeling towards loneliness, the role of religion, legal framework towards gender equality etc. Have you think about including some of these?

→ As suggested, We have briefly added some of these background information in Introduction.

METHODOLOGY

4. I would like to know if you have studied the impact of some variables such as:

a. The area: rural or urban. I suppose the prefectures and municipalities may have both areas, if not please correct me.

→ In general, instead of rural / urban, municipalities are coded from 1 (metropolitan area) to 6 (remote area with the lowest level of population density) in official surveys. However, this information was deleted in the dataset used in the study for the ethical purpose.

b. The access to services

→ As the survey questionnaire did not include many parts of related information on the access to services, we were not able to analyze them.

c. The kind of home: flat, detached, semi-detached

→ We added variable on home ownership in the model.

d. Employment status

→ This variable has been added in the model.

e. Do respondents receive help from the public institutions, cash transfers etc?

→ The study was implemented by staffs of local health centers. To each respondent, a small gift instead of cash transfer was given.

5. Why have you decided to dichotomize the answers into “strongly agree / somewhat agree versus strongly disagree / somewhat disagree”. Have you thought about the relationship between “somewhat agree” and “somewhat disagree”? The real feeling behind both answers can be determined by other factors while the “extreme” options can rely on stronger arguments

→ The reason of dichotomizing was to avoid potential small size in disagree items to preform multilevel regression models, while as suggested we added a full description of the four items to capture diverse answers.

6. Have you tried another options instead of creating age groups? Have you tried to analyze the nonlinear relationship between age and the answers?

→ Yes, we had tried several options to create age groups and analyzed the nonlinear relationship between age and the target outcome and got the three-level age variable. It is able to explain the difference by age following the social context of Japan.

7. It could be interesting to include more participants’ information from the gender perspective to compare the characteristics of both groups.

→ The model included related variables in the questionnaire to capture the characteristics of both groups. As suggested above, in the revision, we have added employment status in the model to adjust the predict the overall proportion of the positive answer (it was not significant, though).

RESULTS AND FINDINGS

8. Regarding the women/men comparison: Why do you report results on household income for men but the financial support for women? (This is just an example on two variables, but the comment could be extended to others) Maybe this can be explained by the role women play in Japan. Then, don’t you think women could not feel comfortable with this role and this can affect their feelings towards ageing?

→ Based on the results as showed in Table 4, middle household income and no household savings in men are less likely to have positive attitude compared to alternatives but income and household savings in women did not have the significant impact. Rather, those not qualified to public assistance in women have higher proportion of the negative answer. It may reflect the fact that men’s attitude to long-live depends on earnings, while women’s attitude may be attributable to being financially secured, slightly different. To some extent it suggests the “ongoing” but not “completed” transition of productive and reproductive roles of men and women. Regarding women’s attitude, their proportion of the positive answer were lower compared to men.

9. The feeling towards ageing may vary with age. Are these variations the same for men and women? If they differ, how could you explain it?

→ The descriptive results indicated that the anticipation to longevity tended to decrease with age in general, while in multilevel analysis, age is significant in women, as those aged between 40-70 years are less likely to have the positive answer, but isn’t significant in men. Considering the social context in Japan, this may be explained by the fact that women in the age group 40-70 years play a major role in nursing care for the elderly, as discussed in our paper.

10. Authors focus on the health status to explain the gender gap. I think that in this case, other factors are influencing respondents’ answers. Authors mention that “contextual influences” or “personal beliefs” are also relevant according to the literature. How do you think the women participation in the labor market could affect their attitude towards ageing?

→ Based on the findings, we discussed the aspects of health, home-based caregiving roles, social support and financial status. We added employment status in the model as suggested (it was not significant, though).

Regarding the impact of women’s participation in the labor market, though it’s not directly showed in our results due to complicated mechanisms, it potentially influences the social value on aging in various aspects. For example, at the macro level, it may improve women’s financial status, lead to a profound change in marriage and family pattern, in social network and in gender role on nursing care. Some of these may be positive while others may be negative to longevity.

to publish the peer review history of their article ([what does this mean?](https://journals.plos.org/plosone/s/editorial-and-peer-review-process#loc-peer-review-history)). If published, this will include your full peer review and any attached files.

If you choose “no”, your identity will remain anonymous but your review may still be made public.

**Do you want your identity to be public for this peer review?** For information about this choice, including consent withdrawal, please see our [Privacy Policy](https://www.plos.org/privacy-policy).

Reviewer #1: No
