# Supplementary material for: Gender disparity in the individual attitude toward longevity among Japanese population: Findings from a national survey
Source: PLoS One. 2021 Aug 18;16(8):e0254779. doi: 10.1371/journal.pone.0254779 (PMC8372936; doi:10.1371/journal.pone.0254779)
Supplement: Attachment — Submitted filename: PLoS One_response to reviewers R2.docx [file pone.0254779.s003.docx]

**Response to Journal Requirements:**

Please review your reference list to ensure that it is complete and correct. If you have cited papers that have been retracted, please include the rationale for doing so in the manuscript text, or remove these references and replace them with relevant current references. Any changes to the reference list should be mentioned in the rebuttal letter that accompanies your revised manuscript. If you need to cite a retracted article, indicate the article’s retracted status in the References list and also include a citation and full reference for the retraction notice.

→ The reference list has been checked.

**Response to Reviewers' comments:**

Comments to the Author

1. If the authors have adequately addressed your comments raised in a previous round of review and you feel that this manuscript is now acceptable for publication, you may indicate that here to bypass the “Comments to the Author” section, enter your conflict-of-interest statement in the “Confidential to Editor” section, and submit your "Accept" recommendation.

Reviewer #1: (No Response)

2. Is the manuscript technically sound, and do the data support the conclusions?

The manuscript must describe a technically sound piece of scientific research with data that supports the conclusions. Experiments must have been conducted rigorously, with appropriate controls, replication, and sample sizes. The conclusions must be drawn appropriately based on the data presented.

Reviewer #1: Yes

3. Has the statistical analysis been performed appropriately and rigorously?

Reviewer #1: Yes

4. Have the authors made all data underlying the findings in their manuscript fully available?

The [PLOS Data policy](http://www.plosone.org/static/policies.action#sharing) requires authors to make all data underlying the findings described in their manuscript fully available without restriction, with rare exception (please refer to the Data Availability Statement in the manuscript PDF file). The data should be provided as part of the manuscript or its supporting information, or deposited to a public repository. For example, in addition to summary statistics, the data points behind means, medians and variance measures should be available. If there are restrictions on publicly sharing data—e.g. participant privacy or use of data from a third party—those must be specified.

Reviewer #1: No

5. Is the manuscript presented in an intelligible fashion and written in standard English?

PLOS ONE does not copyedit accepted manuscripts, so the language in submitted articles must be clear, correct, and unambiguous. Any typographical or grammatical errors should be corrected at revision, so please note any specific errors here.

Reviewer #1: Yes

6. Review Comments to the Author

Please use the space provided to explain your answers to the questions above. You may also include additional comments for the author, including concerns about dual publication, research ethics, or publication ethics. (Please upload your review as an attachment if it exceeds 20,000 characters)

Reviewer #1: The new version has included some improvements following the recommendations of reviewers. Nevertheless, there are still some comments to be made.

1. One of the comments regarding the dataset was the inclusion of a variable capturing the type of area (rural or urban). Authors argue the existence of a variable (coded 1-6) that captures this fact. Nevertheless, they have not included it due to “ethical concerns”. I do not understand the reason why including information about the level of population in an area would mean a problem with ethics.

→ An ethical concern refers to the fact that after cross calculations, the size in the category 6 region (the remote area with the lowest level of population density) may be too small to remain anonymization in reality.

2. What are the changes in the model and results after including some of the recommended variables?

→ As recommended, we added home ownership and employment status in the regression models and the results were not affected in the overall population and by gender (Table 3 and Table 4).

3. Another important comment referred to the decision of dichotomize the answers into “strongly agree / somewhat agree versus strongly disagree / somewhat disagree”. Authors have included information without dichotomizing this variable. Authors argue the “potential small size” in some items. I cannot fully agree with this issue: 1. Strongly agree (N=4816), 2. Somewhat agree (N=8634), 3. Somewhat disagree (N=5287) and 4. Strongly Disagree (N=801). I still thing that dichotomizing maybe is not the best approach. Could you please explain it better, give more reasons for this or include some other studies that have performed the analysis in the same way?

→ Apologies for deficient explanations. Regarding whether or not dichotomizing the answers, our concern is that statistical power would be weakened when cross calculating the answer of the longevity desire with a number of variables. For example, when cross-calculating the distribution of the answer in those qualified for the public assistance (a total of 237) by gender, some items end up with less than 5. Still, we realize the overview of the participants and the survey would be benefit from the advice, so we added the distribution of the answers without dichotomizing in Table 2.

4. I think that including changes in the manuscript including the information and the answers to the comments would be useful for future readers that may have similar doubts/concerns when reading you interesting paper.

→Based on the advice, we added “The trajectory of essential family functions in caregiving of the elderly people is being weakened”, as marked light green in Discussion. The major information from the Discussion were following the finds.

7. PLOS authors have the option to publish the peer review history of their article ([what does this mean?](https://journals.plos.org/plosone/s/editorial-and-peer-review-process#loc-peer-review-history)). If published, this will include your full peer review and any attached files.

If you choose “no”, your identity will remain anonymous but your review may still be made public.

**Do you want your identity to be public for this peer review?** For information about this choice, including consent withdrawal, please see our [Privacy Policy](https://www.plos.org/privacy-policy).

Reviewer #1: No
