# Supplementary material for: Gender disparity in the individual attitude toward longevity among Japanese population: Findings from a national survey
Source: PLoS One. 2021 Aug 18;16(8):e0254779. doi: 10.1371/journal.pone.0254779 (PMC8372936; doi:10.1371/journal.pone.0254779)
Supplement: Attachment — Submitted filename: Response to comments_20210521.docx [file pone.0254779.s004.docx]

**Response to comments**

We highly appreciated the reviewer’s comments, which have contributed to the improvement of our work. Meanwhile, let me express apologies for bad clarifications on variable handling and result interpretation, causing misunderstanding and confusions. Please kindly find our response one-by-one as below.

Reviewer #1: I have to make some comments to this manuscript. As I said in previous stages, the subject the paper addresses is very interesting. Nevertheless, I feel that authors has not been able to address properly all my comments:

1. Regarding ethics: Ethics can be understood as an important concern when talking about individuals. I have several doubts about applying ethical concerns due to anonymization for regions.

→ My apologies for bad clarifications, causing misunderstanding. The ethical concerns remain when the size in some cross calculations (in particular those living in the remote region) is relevantly small, potentially causing a risk to get the individual identified directly or indirectly by reference to identifiers such as the location and demographical / socioeconomic factors.

For example, when cross calculating the regions × basic factors and getting a relevantly small number in a cell, it turned out that, it would be possible to trace or predict a natural person who took part in the survey in the setting by referring these factors (age, annual income, house ownership, public assistance, etc), even though the individual had been anonymized. This issue is in particular taken prudently when handling this kind of government survey / statistics.

For this reason, in the procedure for the application to the secondary survey data use, the regional variable were not included into the dataset for this study after all.

2. Binary variables: It is true that problems can arise due a little N. I do not know whether authors have tried another approach for these variables. Moreover, I wonder why not including for example the variable rural vs urban using the coding 1-6 that is available by transforming these codes into 0-1?

　→Regarding the dependent variable, as the reviewer suggested dichotomizing may not be the best approach, we once considered sticking with the original variable, while our concern remains in that statistical power would be weakened due to a little N. For this reason, it may be worthy to keep dichotomizing.

As for variable “rural and urban”, I understand the reviewer recommended adding it, which is quite common in practice. However, I am afraid that this time we could not do so in the analysis, as the regional variable hadn’t been included into the dataset we used for this study when applying for the secondary analysis because of the ethical concern as mentioned above. My apologies for making confusions.

3. Recommended variables: Authors state that they have included “home ownership” and “employment status” and final results were not affected. Nevertheless, authors have not included these regressions in the paper or an explanation of the reasons behind the fact described by them (new variables do not affect the dependent variable). The attitude towards of longevity is the same for a young person who has never worked than for a similar person who has always have a well-paid job?

→As recommended, we further included home ownership and employment status to the regression model as the explanatory variables (as briefly described in Method) and their results were added in Table 3 (for the whole population) and Table 4 (for each male and female group). Although in the univariate analysis, the results suggested a significant association between the two new variables and the target variable, the multilevel analysis results indicated that these two variables were no longer significantly affecting the attitude to longevity, after adjusting confounding effects of those variables input in the model. Then based on the results, those showed significance (p<0.05) were considered to have an independent influence to the target variable, and consequently discussed and examined in Discussion.

Regarding whether “the attitude to longevity is the same for a young person who has never worked than for a similar person who has always have a well-paid job”, it is worthy note that our results need to be interpreted at the population level and in a cross-sectional perspective, but have limited power to predict the attitude at the individual level and in a longitudinal perspective.

It may be rational to assume that a young person who has never worked is probably not having a positive desire compared to his/her counterpart with a well-paid job at the individual level, while looking at young persons as a group, those with middle-level income and those not qualified to the public assistance tended to be less likely to have a positive desire while employment status didn’t show a significant influence (it’s not to say the employment isn’t related to the attitude to longevity, but its effect is going to be diminished when adjusting confounding effects in the multilevel model). As mentioned in Discussion, these findings may be explainable by the fact those with low-level income and qualified for the public assistance are publicly subsidized for healthcare and long-term care, that the income level in the middle class is lowering, and that inequalities are widening as the results of increasing temporary employment, job polarization and population aging. It suggests concerns on the late life arising from the changing social context of Japan, though due to the cross-sectional design as mentioned in the limitations (the last paragraph of Discussion), these kinds of longitudinal effects cannot be simply captured by the current survey.
